# Engineering *E. coli*–*E. coli* cocultures for production of muconic acid from glycerol

**DOI:** 10.1186/s12934-015-0319-0

**Published:** 2015-09-15

**Authors:** Haoran Zhang, Zhengjun Li, Brian Pereira, Gregory Stephanopoulos

**Affiliations:** Chemical Engineering Department, Massachusetts Institute of Technology, 77 Massachusetts Ave, Cambridge, MA 02139 USA; Beijing Key Laboratory of Bioprocess, College of Life Science and Technology, Beijing University of Chemical Technology, Beijing, 100029 People’s Republic of China

**Keywords:** Metabolic engineering, *E. coli*, Coculture, Muconic acid, Glycerol

## Abstract

**Background:**

*cis*, *cis*-Muconic acid is an important chemical that can be biosynthesized from simple substrates in engineered microorganisms. Recently, it has been shown that engineering microbial cocultures is an emerging and promising approach for biochemical production. In this study, we aim to explore the potential of the *E. coli*–*E. coli* coculture system to use a single renewable carbon source, glycerol, for the production of value-added product *cis*, *cis*-muconic acid.

**Results:**

Two coculture engineering strategies were investigated. In the first strategy, an *E. coli* strain containing the complete biosynthesis pathway was co-cultivated with another *E. coli* strain containing only a heterologous intermediate-to-product biosynthetic pathway. In the second strategy, the upstream and downstream pathways were accommodated in two separate *E. coli* strains, each of which was dedicated to one portion of the biosynthesis process. Compared with the monoculture approach, both coculture engineering strategies improved the production significantly. Using a batch bioreactor, the engineered coculture achieved a 2 g/L muconic acid production with a yield of 0.1 g/g.

**Conclusions:**

Our results demonstrate that coculture engineering is a viable option for producing muconic acid from glycerol. Moreover, microbial coculture systems are shown to have the potential for converting single carbon source to value-added products.

## Background

*cis*, *cis*-muconic acid (MA) is a diunsaturated linear dicarboxylic acid with recognized industrial applications. One of the primary uses of MA is to make adipic acid through hydrogenation [1–4]. As a precursor for the synthesis of several commodity chemicals, such as nylon and polyurethane, adipic acid has a long history of commercial applications in the plastics industry. In addition, MA can be utilized as the starting material for making terephthalic acid, which is one of the two constituent monomers of the high-demand plastic polymer polyethylene terephthalate (PET) [5]. Adipic acid and terephthalic acid also have wide applications in the cosmetic, pharmaceutical and food industries [1]. However, current industrial production of adipic acid and terephthalic acid relies exclusively on utilization of petroleum and coal feedstocks through chemical conversion processes, which has encountered major challenges due to the increasing sustainability, environmental and economic concerns.

Development of new production methods utilizing renewable resources, such as biomass feedstocks, offers more sustainable ways for producing valuable bulk compounds [6]. This has fueled extensive research interest in the engineering of microbes to achieve MA biosynthesis from simple renewable carbon substrates [1]. The use of microorganisms to convert aromatics to MA has been demonstrated before; however, the starting materials used in these reports, such as toluene and benzoic acid, are derived from petroleum or coal raw materials [7–13]. The first complete MA biosynthesis from glucose, a renewable sugar, was established in engineered *E. coli* by the Frost group, which utilized the metabolite 3-dehydroshikimic acid as key biosynthesis precursor [3, 14]. Further optimization of this biosynthetic pathway resulted in 18 g/L MA production from glucose using a fed-batch bioreactor [4]. The same pathway was also employed to biosynthesize MA in the yeast *S. cerevisiae*, although the reported titers and yields of these studies were significantly lower due to the challenge of achieving sufficient functional expression of the heterologous pathway enzymes [15, 16]. In addition, Lin et al. [17] established a new MA biosynthetic route via salicyclic acid and achieved a MA production of 1.5 g/L. The same group also demonstrated a biosynthetic pathway in *E. coli* leading from simple carbon sources to MA via anthranilate [18].

Recently, our group developed a novel metabolic engineering approach based on a coculture concept [19] and successfully utilized it for achieving high-yield MA biosynthesis from sugar mixtures that can be derived from lignocellulose [20]. In the present study, we further explore the potential of the microbial coculture approach by using a single carbon source, glycerol, to biosynthesize MA. Although *E. coli*–*E. coli* cocultures have been utilized to produce small molecules, such as lactic acid, previous studies focused on utilizing different carbon sources [21, 22]. Other studies engineered *E. coli* cocultures for the production of more complex molecules. For example, Saini et al. [23] employed an *E. coli*–*E. coli* coculture for biosynthesis of n-butanol from sole carbon source glucose. The novelty of the present study is the use of cocultures to balance metabolic pathways harboring a very slow step that leads to secretion of pathway intermediates and, as a result, low product yields. By using the coculture consisting of two different strains, the low activity of an enzyme can be overcome by increasing the relative amount of the strain harboring the slow step.

On the other hand, we aimed to produce value-added MA from the renewable and inexpensive glycerol substrate. As a major byproduct of the biodiesel industry (roughly 10 % w/w), crude glycerol is produced in surplus amounts relatively to the global market demand. Moreover, disposal of glycerol can cause environmental issues and thus needs to meet regulatory requirements that increase the operational cost of biodiesel production. Nevertheless, glycerol can be efficiently utilized by a variety of microbes for growth, and the related biodegradation pathways have been well studied. It is therefore of great research and industrial significance to develop new bioprocesses that convert glycerol to valuable compounds [24–26]. This study reports the conversion of the substrate glycerol to *cis*, *cis*-muconic acid using engineered *E. coli*–*E. coli* cocultures.

## Results and discussion

### MA synthesis from glycerol using an *E. coli* monoculture

**Fig. 1:**
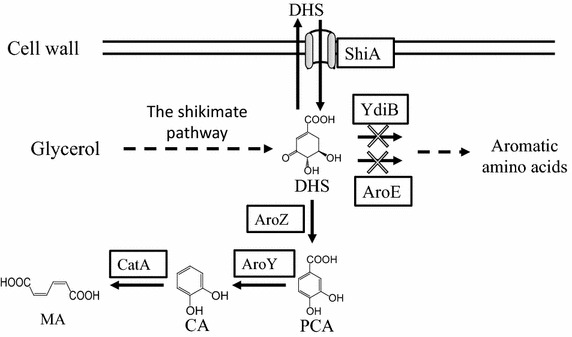
Biosynthetic pathway of muconic acid in *E. coli*. *DHS* 3-dehydroshikimic acid, *PCA* protocatechuic acid, *CA* catechol, *MA* *cis*, *cis*-muconic acid. *Boxed letters* represent pathway enzymes

Several MA biosynthetic pathways have been assembled in heterologous microorganisms for de novo MA synthesis from simple carbon substrates [4, 17, 18]. In this study, we chose to use the biosynthetic route that makes MA through three enzymatic reactions via intermediates 3-dehydroshikimic acid (DHS), protocatechuic acid (PCA) and catechol (CA) (Fig. 1). Heterologous enzymes DHS dehydratase (AroZ) and PCA decarboxylase (AroY) from *Klebsiella pneumonia* and CA 1,2-dioxygenase (CatA) from *Acinetobacter calcoaceticus* were used to establish this pathway [4, 20]. An *E. coli* tyrosine over-producer was selected to accommodate the MA biosynthetic pathway, as this strain has an engineered shikimate pathway to efficiently produce the required DHS intermediate. Two competing genes, including *ydiB* and *aroE*, were deleted from the *E. coli* chromosome to yield strain P5 that eliminated the undesired conversion of DHS to the shikimate pathway downstream products. The introduction of the MA biosynthetic pathway in *E. coli* P5 yielded strain P5g. When MA biosynthesis was induced at the beginning of cultivation, it was observed that P5g grew poorly on glycerol, indicating that overexpression of the pathway enzymes imposed significant metabolic stress on *E. coli* and thus impaired its growth. This issue was addressed by the delayed addition of the inducer IPTG 24 h after inoculation. Under this condition, P5g grew better and was able to make 316 mg/L MA from 10 g/L glycerol. Consistent with our previous finding [20], a high titer of the intermediate DHS accumulated in the medium, suggesting that availability of intracellular DHS could be limiting MA production.

**Fig. 2:**
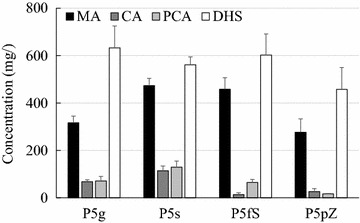
MA production by monoculture engineering strategies. P5g, *E. coli* with the engineered shikimate pathway and reconstituted MA biosynthetic pathway. P5s, P5g overexpressing the ShiA permease. P5fs, P5g with fusion expression of AroZ and ShiA. P5pZ, P5g with AroZ localized in the periplasmic space. *Dark column* MA, *dark grey* CA, *grey* PCA, *white* DHS. *Error bars* are SEM obtained from at least three independent experiments

It has been reported that an endogenous *E. coli* membrane-bound transporter, ShiA permease, can mediate DHS trans-membrane transport and, as such, can be utilized for DHS assimilation [20]. Hence, the *shiA* gene was over-expressed in *E. coli* to improve DHS importation and increase the intracellular DHS-to-MA conversion. As shown in Fig. 2, this strategy indeed increased MA production to 473 mg/L in the resulting strain *E. coli* P5s, accompanied by a slight decrease in total DHS accumulation.

In an effort to further improve DHS conversion, the downstream pathway enzyme AroZ, responsible for the DHS-to-PCA conversion, was fused to the ShiA permease so that imported DHS could be immediately processed by AroZ to produce the intermediate PCA before leaking out of the cell again. However, *E. coli* P5fs constructed using this design did not show any significant improvement of MA production (Fig. 2). In another construct, the AroZ enzyme was engineered to be localized in the periplasmic space between the *E. coli* inner and outer membranes through the use of a phage gIII capsid protein signal sequence [27, 28]. In this construct, imported DHS would not need to travel across both cell membranes to gain access to the cytosolic AroZ enzyme for downstream conversion, which could potentially alleviate any DHS cross-membrane transportation issues and improve downstream conversion efficiency. However, this strategy turned out to be unsuccessful and actually resulted in decreased MA production (Fig. 2). The reason could be that the periplasmic expression of AroZ generated a physiological impact on cell’s fitness and decreased the cell’s production performance.

### MA production by a coculture of *E. coli* P5.2 and BLS

The lack of MA production improvement by the above metabolic engineering efforts suggested that alternative methods should be deployed in order to reduce DHS accumulation. To this end, we employed an *E. coli*–*E. coli* coculture engineering approach to improve MA biosynthesis from glycerol. In fact, a previous report demonstrated a coculture system for converting sugar mixtures to the MA product [20]. Here, we further explored the potential of coculture engineering to utilize a single carbon source, glycerol.

**Fig. 3:**
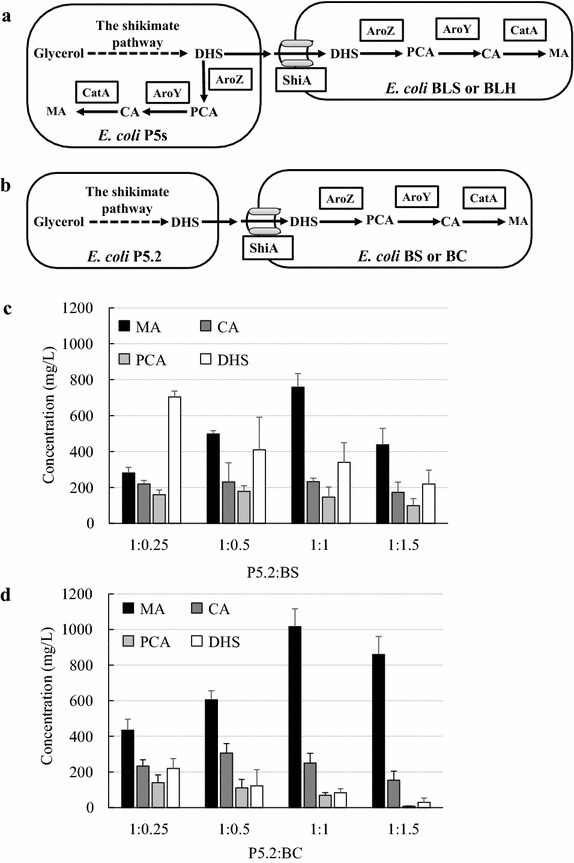
MA production by utilization of *E. coli*–*E. coli* cocultures. **a** Schematic of the coculture design to use a second cell BLS or BLH to work with P5s for MA production. **b** Schematic of the coculture design to split the complete biosynthetic pathway between upstream P5.2 and downstream BS or BC cells. **b** Production optimization by varying the inoculation ratio of P5.2 and BS. **c** Production optimization by varying the inoculation ratio of P5.2 and BC. *Error bars* are SEM obtained from at least three biological replicates

Considering that there was still a high level of DHS intermediate accumulation in the *E. coli* P5s monoculture, we next supplemented a second *E. coli* strain overexpressing the heterologous enzymes AroZ, AroY and CatA and the ShiA permease to better utilize the secreted DHS (Fig. 3a). This strain, named BLS, was in fact derived from *E. coli* BL21(DE3) that had been found to better support heterologous enzyme activity for the MA biosynthetic pathway [20]. It should be noted that *E. coli* BLS did not have an engineered shikimate pathway and thus could only utilize DHS secreted from P5s for MA biosynthesis. When P5s and BLS were co-cultivated, the MA production showed varied profiles depending on the inoculation ratio (Table 1). When the P5s:BLS inoculation ratio was decreased by the addition of more BLS in the coculture system, the DHS conversion capacity was improved. Accordingly, DHS decreased while MA concentration increased. However, there is a maximum in the amount of the BLS strain in the inoculum beyond which DHS conversion to MA was not improved, as the relative population size of P5s in the coculture was repressed for providing the DHS intermediate for MA formation. These constructs yielded a highest MA production of 639 mg/L and yield of 0.064 g/g at an inoculation ratio of 1:1. However, despite improvement of MA production, DHS was still not completely converted in the P5s:BLS coculture system.

**Table 1:** Production of MA using P5s:BLS coculture

		Product and intermediate concentrations (mg/L)				Yield (g/g)
		MA	CA	PCA	DHS	
	P5s	473 ± 31	114 ± 20	130 ± 26	562 ± 33	0.047
P5s:BLS inoculation ratio	1:0.25	416 ± 33	160 ± 48	92 ± 17	223 ± 33	0.042
	1:0.5	562 ± 52	114 ± 22	95 ± 18	194 ± 15	0.056
	1:1	639 ± 83	230 ± 38	69 ± 12	198 ± 14	0.064
	1:1.5	418 ± 80	152 ± 39	78 ± 30	164 ± 31	0.042
	BLS	26 ± 2	8 ± 5	0.2 ± 0.2	1.1 ± 0.7	0.003

### Utilization of *E. coli* chaperones in the P5s:BLH coculture system

Next, we attempted to enhance the DHS-to-MA conversion by improving the heterologous enzymes’ activity with the help of *E. coli* chaperones. Specifically, a new *E. coli* strain BLH was constructed to over-express the *E. coli* GroEL/GroES chaperones as well as the enzymes AroZ, AroY, CatA and ShiA. When BLH was co-cultivated with P5s, DHS accumulation was also reduced, similar to what was observed for the P5s:BLS coculture, as shown in Table 2. Optimization of the inoculation ratio of P5s:BLH resulted in a production of 691 mg/L MA and 255 mg/L DHS. Under the optimal inoculation ratio, the MA product concentration and yield were both comparable to what was achieved by the P5s:BLS coculture, indicating that addition of *E. coli* chaperone systems did not make a significant impact on the MA biosynthesis. Notably, the optimal inoculation ratios of P5s:BLS and P5s:BLH are different, which could be due to the varied growth rate and DHS-to-MA conversion capacity between BLS and BLH.

**Table 2:** Production of MA using P5s:BLH coculture

		Product and intermediate concentrations (mg/L)				Yield (g/g)
		MA	CA	PCA	DHS	
	P5s	473 ± 31	114 ± 20	130 ± 26	562 ± 33	0.047
P5s:BLH inoculation ratio	1:0.25	591 ± 57	182 ± 50	143 ± 3	238 ± 32	0.059
	1:0.5	691 ± 112	189 ± 76	114 ± 25	255 ± 42	0.069
	1:1	488 ± 55	77 ± 26	47 ± 3	144 ± 24	0.049
	1:1.5	480 ± 3	79 ± 10	109 ± 26	123 ± 15	0.048
	BLH	128 ± 21	190 ± 56	33 ± 6.0	3.2 ± 2.5	0.013

Despite different engineering strategies to improve DHS conversion in the monoculture strategy, such as utilization of the ShiA transporter, fusion expression of ShiA and AroZ, and periplasmic expression of AroZ, DHS accumulation was not completely eliminated in engineered *E. coli*. In contrast, when a second *E. coli* strain containing the DHS-to-MA pathway was introduced into the production process, more DHS conversion capacity was provided and DHS was better converted to the final MA product. In both cases of P5s:BLS and P5s:BLH cocultures, the benefit of increasing DHS-to-MA conversion outweighed the disadvantage of introduced growth competition between the two constituent strains. As a result, significant MA production improvement was observed.

### Splitting the MA biosynthesis pathway between two strains cultivated in coculture

We next split the entire MA biosynthetic pathway into two modules, each of which was accommodated in a separate *E. coli* strain. The upstream strain *E. coli* P5.2 contained only the shikimate pathway ending with the synthesis of DHS, whereas the downstream strain *E. coli* BS was equipped with enzymes AroZ, AroY, CatA and ShiA under the control of the inducible T7 promoter to assimilate and convert DHS to MA. The overall design of this new coculture strategy is shown in Fig. 3b.

Consistent with previous observations, MA production depended on the inoculation ratio of P5.2 and BS (Fig. 3c). The highest MA production of 758 mg/L was achieved at the inoculation ratio of 1:1, indicating that the upstream and downstream pathways were best balanced for the complete glycerol-to-MA biosynthesis process under this condition. Although the DHS concentration gradually decreased as more BS cells were supplemented in the cocultures, DHS accumulation was still observed even at the highest inoculation ratio. This finding suggested that inducible expression of the enzymes responsible for DHS to MA conversion was not the best strategy for eliminating DHS accumulation. In fact, it was observed before that use of a milder constitutive expression strategy helped improve MA biosynthesis [20]. Therefore, we constructed a new downstream strain *E. coli* BC that over-expressed the downstream pathway enzymes and the ShiA permease under the control of a constitutive pyruvate decarboxylase promoter isolated from *Zymomonas mobilis*. It should be noted that the upstream strain P5.2 also used constitutive promoters for DHS biosynthesis and thus the new P5.2:BC coculture system did not need any inducer for MA biosynthesis.

As shown in Fig. 3d, MA production with the new coculture system of P5.2:BC was improved at all inoculation ratios. At the optimal inoculation ratio of 1:1, MA concentrations reached 1016 mg/L. Moreover, DHS accumulation was also further reduced in this new coculture system. Notably, constitutive expression of the entire biosynthetic pathway in a single strain system, *E. coli* P5cS, resulted in poor growth and reduced MA production (56 mg/L), due to overwhelming metabolic burden imposed by consolidated over-expression of all pathway enzymes. These findings together suggest that lessening the metabolic stress on the microbial host improved its fitness for functional expression of the heterologous downstream enzymes; and the production improvement shown here highlights the importance and usefulness of the coculture strategy to address this issue.

**Fig. 4:**
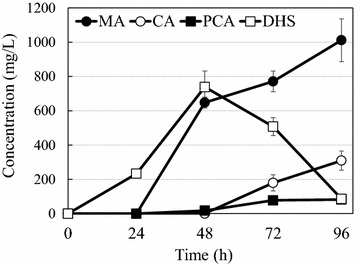
Time profile of the MA product and intermediates concentration changes in the P5.2:BC coculture system. *Error bars* are SEM obtained from three independent experiments

We further analyzed the dynamic production profile during the P5.2:BC coculture cultivation process. As shown in Fig. 4, the DHS concentration peaked at 48 h, followed by a significant decrease in the next 48 h, indicating that the downstream DHS conversion capacity provided by BC exceeded the upstream DHS formation capacity after 48 h. In comparison, accumulation of PCA and CA intermediates was maintained at relatively low levels, although their concentration started to increase toward the end of the production process. The MA final product concentration stably increased with time over the whole co-culturing process.

### Bioreactor production

**Fig. 5:**
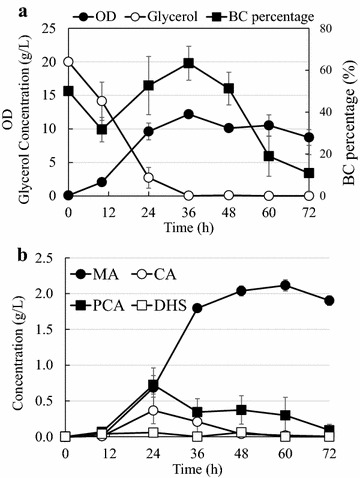
Bioreactor production of MA using the P5.2:BC coculture system. **a** The coculture cell density, BC percentage and glycerol titer changes during the cultivation process. **b** The MA and pathway intermediate concentration changes during the cultivation process. *Error bars* represents SEM obtained from three independent bioreactor runs

We next attempted to further increase MA biosynthesis by scaling up the coculture production process in a batch bioreactor. Specifically, *E. coli* P5.2 and BC were co-cultivated on 20 g/L glycerol at the inoculation ratio of 1:1. As shown in Fig. 5a, the overall cell density of the P5.2:BC coculture reached ca. OD_600_ = 10 after 24 h, and stabilized at this level until the end of cultivation. The glycerol substrate was rapidly consumed at the early stage of the bioreactor production, and depleted at 36 h. As the two constituent strains in the coculture competed for glycerol as carbon source, the growth interaction between them was highly dynamic. In fact, BC percentage in the coculture population fluctuated around 50 % in the first 36 h. A significant decline of BC percentage was then observed after 36 h, which should be due to the relative poor growth of BC expressing the heterologous enzymes upon the depletion of the glycerol carbon source.

The MA and intermediate production profiles are shown in Fig. 5b. The concentrations of intermediates CA, PCA and DHS were repressed throughout the cultivation process, indicating that these intermediates were well converted to downstream products in the downstream strain BC. The MA concentration increased rapidly within the first 36 h, and then plateaued at 2 g/L due to depletion of the glycerol substrate. The highest production yield of 0.1 g/g is similar to the results achieved in the previous section. The development of the production curves in Fig. 5b demonstrates that the intermediate-to-MA conversion capacity was reduced after 36 h, which is consistent with the decline of BC sub-population caused by the glycerol depletion.

The theoretical maximum yield for the pathway leading from glycerol to MA is calculated as 0.66 g/g, using the equation for pathway balance 7 Glycerol + 4 ATP + 10 NAD = 3 MA + 3 CO_2_ + 4 ADP + 10 NADH. Although carbon loss for biomass formation and respiration is not considered, the achieved yield is significantly lower than the theoretical maximum, indicating that there is room for further engineering efforts to improve MA production yield.

## Conclusions

We report a coculture engineering approach to convert renewable glycerol substrate to an important value-added product. Through step-wise engineering and optimization efforts, we significantly improved the product concentration and yield. We also demonstrate that, despite growth competition between the two strains, the *E. coli*–*E. coli* coculture can be used in the context of sole carbon source for complex biosynthesis pathway engineering. The results of this study indicate that the combination of pathway modularization and microbial co-cultivation has strong potential for future metabolic engineering studies.

## Methods

### Plasmid and strain construction

Gene cloning and bacterial transformations were carried out according to the standard protocols [29]. PCR primers were purchased from Lifetechnologies (Grand Island, NY, USA). Phusion DNA Polymerase for PCR amplification endonucleases, and the Gibson assembly kit used in this study were purchased from New England Biolabs (Ipswich, MA, USA). Chaperone expression plasmid pG-KJE was purchased from Clontech Laboratories (Mountain View, CA, USA).

Some of the plasmids and strains used in this study were constructed in our previous research [20]. Additionally, we constructed several plasmids and *E. coli* strains for MA production from glycerol. To construct plasmid pYfsZ, the *shiA* gene was PCR amplified using primers GAGAAAAACCAAGGCAGTGCCAA GTGAAGCTTTCAAGGAGGAACAGACATGG and CGCTGCCGCTACCAG CGCGTTGACTGTCTTTC; the *aroZ* gene was PCR amplified using primers CAGTCAACGCGCTGGTAGCGGCAGCGGTAGCGGTAGCGGCAGCCTGCGTA GCATTGCCAC and ATCTCAGTGGTGGTGGTGGTGGTGCTTAACAGTACTG CATTGCTGCTAAACG. Plasmid pY constructed previously [20] was then linearized with *Spe*I and *Xho*I restriction enzymes and assembled with the *shiA* and *aroZ* genes using the Gibson assembly kit. The resulting plasmid pYfsZ expressed the aroZ and shiA enzyme in the form of a fusion protein through a flexible glycine-serine connecting linker. To construct plasmid pYpZ, the *aroZ* gene was PCR amplified from plasmid pZ [20] using primers CGTCTAGAAAGGAGGAACAGACATGAAAA AACTGCTGTTCGCGATTCCGCTGGTGGTGCCGTTCTATAGCCATAGCATGCTGCGTAGCATTGC (containing a phage gIII capsid protein signal sequence) and CTCGAGGCACTAGTTTAACA. The PCR product was *Xba*I and *Xho*I digested and ligated with plasmid pY digested with the same restriction enzymes. The plasmids were transformed into the desired *E. coli* strains by electroporation. A complete list of the plasmids and *E. coli* strains used in this study is presented in the Table 3.

**Table 3:** The plasmids and *E. coli* strains utilized in this study

Plasmid	Description	Source
pET21c	T7 promoter, Amp^R^	Novagen
pET28a	T7 promoter, Kan^R^	Novagen
pHACM-rpoA14	A gTME plasmid carrying a mutated alpha subunit of RNA polymerase for enhancing the shikimate pathway	[30]
pAO	pET21c carrying the *catA* and auxiliary ACIAD1443 genes from *Acinetobacter calcoaceticus*	[20]
pAOM	pACYCDuet-1 carrying the *catA* gene and ACIAD1443 genes	[20]
pY	pET28a carrying the aroY gene from *Klebsiella pneumoniae*	[20]
pZ	pET28a carrying the *aroZ* gene from *Klebsiella pneumoniae*	[20]
pYZS	pET28a carrying the *aroY* and *aroZ* genes from *Klebsiella pneumonia* and the *E. coli* *shiA* gene	[20]
pYfsZ	pET28a carrying the *aroY* and fused *shiA*-*aroZ* genes	This study
pYpZ	pY carrying *aroZ* that has a phage gIII leading sequence to localize AroZ enzyme into periplasm	This study
pG-KJE	A plasmid carrying *E. coli* chaperone *dnaK*-*dnaJ*-*grpE* and *groES*-*groEL* genes, Cm^R^ (only *groES*-*groEL* genes were induced for expression)	Clontech
pII	A low copy number plasmid expressing weak green fluorecence protein, Kan^R^	[31]
pCS	pACYCDuet-1 carrying the *shiA* gene that is under the control of a constitutive lacuv5 promoter	[20]
pCS2	pCDFDuet-1 carrying the *shiA* gene that is under the control of a constitutive lacuv5 promoter	[20]
pCYZAO	pUC57(Kan) carrying the *aroY*, *aroZ*, *catA* and ACIAD1443 genes that are under the control of a constitutive *Zymomonas mobilis* pyruvate decarboxylase promoter	[20]

Strain	Description	Source
P5	*E. coli* K12 Δ*pheA* Δ*tyrR* *lacZ*::P_LtetO-1_-*tyrA* ^*fbr*^ *aroG* ^*fbr*^ *tyrR*::P_LtetO-1_-*tyrA* ^*fbr*^ *aroG* ^*fbr*^ *hisH*(L82R) Δ*aroE* Δ*ydiB*	[20]
P5g	P5 harboring pHACM-rpoA14, pAO and pYZ	[20]
P5s	P5 harboring pHACM-rpoA14, pAO and pYZS	[20]
P5fs	P5 harboring pHACM-rpoA14, pAO and pYfsZ	This study
P5pZ	P5 harboring pHACM-rpoA14, pAO and pYpZ	This study
P5cS	P5 harboring pHACM-rpoA14, pCYZAO and pCS2	This study
P5.2	P5 harboring pII and pHACM-rpoA14	This study
BLS	*E. coli* BL21(DE3) harboring pHACM-rpoA14, pAO and pYZS	This study
BLH	*E. coli* BL21(DE3) harboring pG-KJE, pAO and pYZS	This study
BS	*E. coli* BL21(DE3) harboring pYZS and pAOM	This study
BC	*E. coli* BL21(DE3) harboring pCYZAO and pCS	This study

### Cultivation conditions

The MA biosynthesis was conducted using a glycerol medium. 1 L glycerol medium contained 10 g glycerol, 2.0 g NH_4_Cl, 5.0 g (NH_4_)_2_SO_4_, 3.0 g KH_2_PO_4_, 7.3 g K_2_HPO_4_, 8.4 g MOPS, 0.5 g NaCl, 0.24 g MgSO_4_, 0.5 g yeast extract, 40 mg tyrosine, 40 mg phenylalanine, 40 mg tryptophan, 10 mg 4-hydroxybenzoic acid, 0.4 mg/L Na_2_EDTA, 0.03 mg H_3_BO_3_, 1 mg thiamine, 0.94 mg ZnCl_2_, 0.5 mg CoCl_2_, 0.38 mg CuCl_2_, 1.6 mg MnCl_2_, 3.77 mg CaCl_2_, and 3.6 mg FeCl_2_. The initial medium pH was adjusted to 6.6 before use. Appropriate antibiotics (100 mg/L ampicillin, 50 mg/L kanamycin, and 34 mg/L chloramphenicol) were also added to the medium when needed. Except for the bioreactor experiments, the MA production was performed in 14 mL culture tubes.

For MA monoculture production, 2 mL glycerol medium was inoculated with 2 % (v/v) overnight *E. coli* P5g or P5s LB culture and cultivated under 37 °C with 250 rpm shaking. Since the addition of IPTG at the time of inoculation resulted in poor cell growth, IPTG was supplemented to the culture 24 h after inoculation to induce the heterologous enzyme expression. MA production was analyzed 72 h after induction.

For MA production using *E. coli*–*E. coli* cocultures, 2 % (v/v) overnight LB cultures of the first strain (i.e. P5s or P5.2) and the second strain (i.e. BLS, BLH, BS or BC) were inoculated into 2 mL glycerol medium and the LB medium, respectively. After 24 h, the optical density (OD) at 600 nm of the two cultures was measured. The second strain was then harvested by centrifugation and appropriate amounts of the cell pellets were re-suspended in the first strain culture to achieve the desired inoculation ratios. To be consistent with the single strain cultivation strategy, 0.1 mM IPTG was supplemented at the time of the second strain addition when *E. coli* BLS, BLH and BS (containing the inducible pathway and transporter enzymes) were used. For P5s:BLH coculture, 5 ng/mL tetracycline was also supplemented at the time of BLH addition to induce the expression of *E. coli* chaperones *groES*/*groEL* carried by plasmid pG-KJE in BLH (the chaperone genes *dnaK*, *dnaJ and grpE* in pG-KJE were not induced for expression). The cocultures were then cultivated under 37 °C with 250 rpm shaking for another 72 h.

For bioreactor cultivation, 14 mL overnight LB culture of *E. coli* P5.2 and appropriate amount (based on their cell densities) of overnight LB culture of *E. coli* BC were inoculated into 0.7 L glycerol medium to achieve 1:1 inoculation ratio. The coculture cultivation was performed in a 1.3-L BioFlo110 modular fermentor system (New Brunswick Scientific) running at 1.5 L/min air flow rate, pH = 7.0 and 37 °C. The agitation rate was controlled to maintain a constant DO of 12 %.

### Assaying the cell-to-cell ratio in the coculture system

The ratio of the two constituent cell types in the *E. coli*–*E. coli* coculture was determined using the method described before [20]. Specifically, 20 μL culture sample taken from the coculture system was first diluted around 10^5^ times using sterile water. 20 μL diluted culture was then spread onto a LB plate containing 40 mg/L X-gal (5-bromo-4-chloro-3-indolyl-β-d-galactopyranoside) and 50 mg/L kanamycin. The plate was then incubated at 37 °C overnight. *E. coli* P5.2 with disrupted *lacZ* gene only formed white colonies on the X-gal plate, whereas the colonies of *E. coli* BC with intact chromosomal *lacZ* gene generated blue color on X-gal. The number of colonies of each color were counted to determine the ratio of the two cell types in the coculture system.

### Substrate and metabolite quantification

Glycerol concentration was monitored by HPLC analysis. Coculture samples were centrifuged at 12,000*g* for 2 min to collect cell-free broth, which was then filtered through 0.2-μm-pore-size polytetrafluoroethylene membrane syringe filters (VWR International) before subjected to HPLC analysis. The HPLC system consisted of a Bio-rad HPX-87H column, a Waters 2695 separation module and a Waters 410 differential refractometer. Isocratic elution was conducted using 14 mM sulfuric acid as mobile phase at a flow rate of 0.7 mL/min.

The concentration of MA product and the pathway intermediates were determined by LC/MS/MS analysis. Cell-free broth was collected by 12,000*g* centrifugation for 5 min followed by filtration through 0.2-μm-pore-size polytetrafluoroethylene membrane syringe filters (VWR International). 10 μL 1 g/L *p*-coumaric acid internal standard was added into 1 mL of the filtered broth. 10 μL of the mixed solution was then injected into an Applied Biosystems API2000 LC/MS/MS running on a previously established method [20].
